# Effect of daphnoretin on the proliferation and apoptosis of A549 lung cancer cells *in vitro*

**DOI:** 10.3892/ol.2014.2296

**Published:** 2014-06-30

**Authors:** HONG-FANG JIANG, ZHUO WU, XUE BAI, YI ZHANG, PING HE

**Affiliations:** 1 Department of Geriatrics, Shengjing Hospital of China Medical University, Shenyang, Liaoning 110004, P.R. China; 2 Department of Thoracic Surgery, The Fourth Affiliated Hospital of China Medical University, Shenyang, Liaoning 110004, P.R. China

**Keywords:** daphnoretin, A549 lung cancer cell, natural compound, apoptosis

## Abstract

Daphnoretin is an active constituent of *Wikstroemia indica* C.A. Mey., which is widely distributed in the northwest and southwest regions of China. Previous studies have shown that daphnoretin has anticancer effects on leukemia, osteosarcoma and uterine cervix cancer cells. However, the effect of daphnoretin on human lung cancer cells has yet to be elucidated. In the present study, daphnoretin was observed to inhibit A549 lung cancer cell proliferation in a concentration- and time-dependent manner. Fluorescent microscopy and flow cytometric analysis showed that daphnoretin induced A549 cell apoptosis in a concentration-dependent manner. Western blot analysis also revealed that daphnoretin induced apoptosis through the regulation of the B-cell lymphoma-2 gene family in A549 cells. These findings indicate that daphnoretin may have potential as a therapeutic agent for the management of lung cancer.

## Introduction

Lung cancer is one of the most frequently diagnosed types of cancer and the leading cause of cancer-associated mortality worldwide (1). Despite the development and use of multimodality therapies, including surgery, radiotherapy, conventional chemotherapy and molecular targeted therapy, the clinical outcome of lung cancer treatment remains unsatisfactory, with a five-year overall survival rate of <15% (2). This may be, at least in part, due to the side-effects associated with currently available chemotherapeutic drugs and the resistance of advanced lung cancer (3). Therefore, novel, effective chemotherapeutic agents are required, particularly those that are derived from natural products due to their intrinsic advantages (4–6).

**Figure 1:**
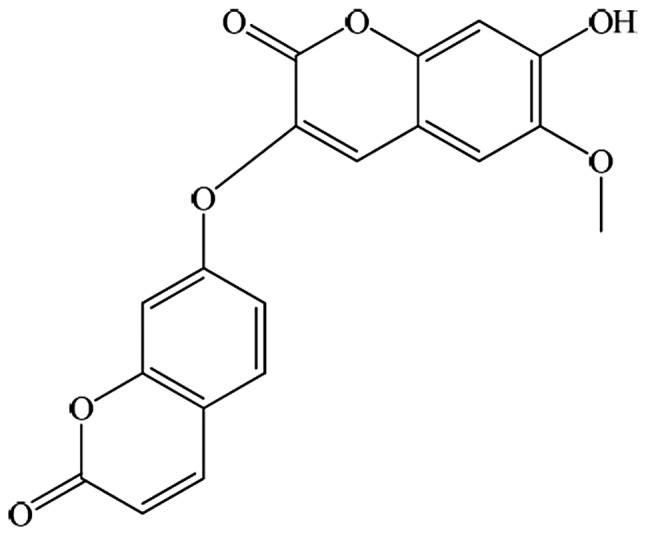
Structure of daphnoretin.

Daphnoretin (Fig. 1) is an active constituent of *Wikstroemia indica* C.A. Mey. which belongs to the Thymelaceae family and is widely distributed throughout the northwest and southwest regions of China. The root of this plant is used as a remedy for arthritis, tuberculosis, syphilis and pertussis (7). Previous studies have shown that daphnoretin has a number of biological activities, including antifungal effects (8), as well as the inhibition of various sites involved in DNA synthesis (9), the activation of protein kinase C in platelet aggregation (7,10), an antiviral effect on hepatitis B (11) and respiratory syncytial virus properties (12). Furthermore, the anticancer effect of daphnoretin has been reported in various studies (13–15). Such studies have shown that daphnoretin exerts its anticancer effects through the inhibition of cancer cell proliferation, the induction of G_2_/M-phase arrest and apoptosis. However, the effect of daphnoretin on human lung cancer cells has yet to be elucidated.

The present study aimed to investigate the effect of daphnoretin on the growth of A549 lung cancer cells and the cellular mechanism involved in daphnoretin-induced apoptosis. The findings of the present study suggest that daphnoretin may have potential as an anticancer agent for lung cancer therapy.

## Materials and methods

### Reagents and chemicals

Daphnoretin was purchased from the National Institute for the Control of Pharmaceutical and Biological Products (Beijing, China) and a 1-mmol/l stock solution of daphnoretin was prepared in dimethyl sulfoxide (DMSO) and stored at −20°C. Deionized water was used in all of the experiments. Fetal bovine serum (FBS) was purchased from Solarbio Science and Technology Co., Ltd. (Beijing, China), and 3-(4,5-Dimethylthiazol-2-yl)-2,5-diphenyltetrazolium bromide (MTT), Hoechst 33342 and DMSO were purchased from Sigma-Aldrich (St. Louis, MO, USA). An Annexin V-fluorescein isothiocyanate (FITC) and propidium iodide (PI) double-staining kit was purchased from KeyGene Inc. (Nanjing, China). Mouse monoclonal anti-human, anti-mouse and anti-rat antibodies against Bcl-2-associated X protein (Bax) (catalogue number, sc-23959), B-cell lymphoma (Bcl)-2 (catalogue number, sc-7382) and glyceraldehyde-3-phosphate dehydrogenase (GAPDH; catalogue number, sc-365062) were obtained from Santa Cruz Biotechnology, Inc. (Santa Cruz, CA, USA). The secondary polyclonal horseradish peroxidase-conjugated goat anti-mouse and anti-rabbit antibodies were also obtained from Santa Cruz Biotechnology, Inc. All other reagents were obtained from Sinopharm Chemical Reagent Co., Ltd. (Shenyang, China).

### Cell culture

The A549 human lung cancer cell line was obtained from the China Center for Type Culture Collection (Wuhan, China) and maintained in RPMI-1640 supplemented with 10% FBS, 100 U/ml penicillin and 100 μg/ml streptomycin at 37°C in a humidified atmosphere of 5% CO_2_.

### MTT assay

The effect of daphnoretin on the proliferation of A549 cells was measured using MTT assay. In brief, A549 cells were plated at a density of 1×10^4^ cells per well on 96-well plates overnight, then treated with various concentrations of daphnoretin (0, 5, 10, 15 and 20 μmol/l) for 24 and 48 h. A total of 20 μl MTT solution [2 mg/ml in phosphate-buffered saline (PBS)] was added to each well and the cells were cultured for 4 h at 37°C. The medium was then removed and 150 μl DMSO was added to solubilize the MTT formazan crystals. The plates were then agitated and the optical density was determined at 570 nm using an ELISA plate reader (Model 550; Bio-Rad Laboratories, Inc., Hercules, CA, USA). At least three independent experiments were performed.

### Fluorescence microscopy

A549 cells (1×10^6^) were seeded on six-well plates overnight, then treated with different concentrations of daphnoretin (0 and 10 μmol/l) for 24 h. The cells were washed twice with cold PBS, fixed with cold methanol and acetic acid (3/1, v/v) for 30 min and then stained with Hoechst 33342 (1 mg/ml) for 30 min in the dark. The stained cells were observed using a fluorescence microscope (magnification, ×400; Nikon E800; Nikon Corporation, Tokyo, Japan).

### Flow cytometric analysis

The apoptotic rates of the A549 cells were determined using flow cytometric analysis using an Annexin V-FITC apoptosis kit. In brief, A549 cells (1×10^6^) were seeded on six-well plates overnight, then treated with various concentrations of daphnoretin (0, 5, 10 and 15 μmol/l) for 24 h. Cells (1×10^6^) were then harvested using centrifugation (326 × g) for 5 min and washed twice with cold PBS. Staining was performed according to the manufacturer’s instructions (KeyGene Inc.) and the cells were analyzed using a FACScan flow cytometer (Becton-Dickinson, San Jose, CA, USA). At least three independent experiments were performed.

### Western blot analysis

The expression of apoptosis-related proteins was assessed using western blot analysis. In brief, A549 cells (1×10^6^) were seeded on six-well plates overnight, then treated with various concentrations of daphnoretin (0, 5, 10 and 15 μmol/l). Following treatment for 24 h, the total proteins were solubilized and extracted using lysis buffer (20 mM HEPES, pH 7.9, 20% glycerol, 200 mM KCl, 0.5 mM EDTA, 0.5% NP-40, 0.5 mM dithiothreitol and 1% protease inhibitor cocktail). The protein concentration was determined using a bicinchoninic acid protein assay. All samples were separated using SDS-PAGE to determine the protein expression of Bax, Bcl-2 and GAPDH. Blots were developed using an enhanced chemiluminescence kit.

### Statistical analysis

Statistical analyses were performed using the SPSS 13.0 package (SPSS, Inc., Chicago, IL, USA). All experiments were performed at least three times. All data are expressed as the mean ± standard deviation. The statistical correlations of the data were tested for significance using analysis of variance and the Student’s t-test. P<0.05 and P<0.01 were considered to indicate statistically significant differences.

## Results

### Daphnoretin inhibits A549 cell proliferation

**Figure 2:**
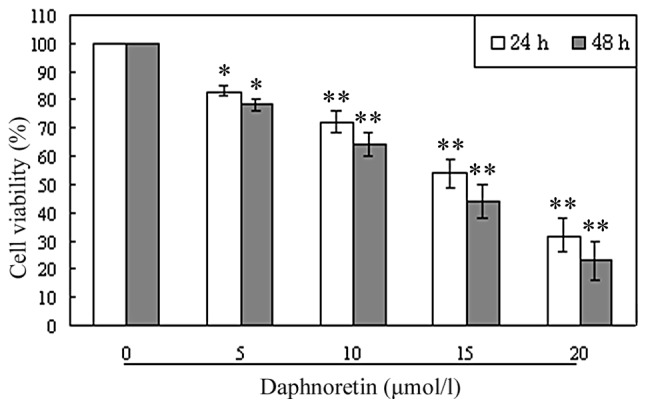
Proliferation-inhibiting effects of daphnoretin on A549 lung cancer cells. ^*^P<0.05 and ^**^P<0.01, vs. the respective control group.

To investigate the growth-inhibiting effect of daphnoretin, A549 cells were treated with various concentrations of A549 for 24 and 48 h, and the rate of inhibition was determined using MTT assay. As shown in Fig. 2, A549 cell growth was observed to be inhibited in a concentration- and time-dependent manner.

### Daphnoretin induces A549 cell apoptosis

**Figure 3:**
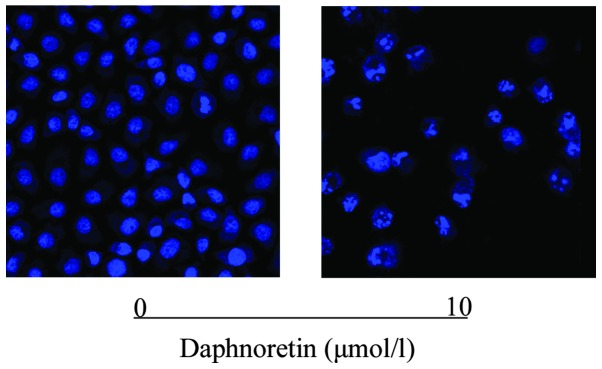
Cell apoptosis observed using Hoechst 33342 staining. A549 cells were treated with daphnoretin (0 or 10 μmol/l) for 24 h. The apoptotic cells exhibited chromatin condensation, nuclear fragmentation and apoptotic bodies.

To investigate the apoptosis-inducing effect of daphnoretin, A549 cells were treated with various concentrations of daphnoretin. Following treatment with daphnoretin (0 and 10 μmol/l) for 24 h, cells were analyzed using fluorescent microscopy with Hoechst 33324 staining. As shown in Fig. 3, chromatin condensation, nuclear fragmentation and apoptotic bodies were observed in the treated cells. The results revealed that when exposed to daphnoretin, A549 cells underwent the typical morphological changes that are associated with apoptosis.

**Figure 4:**
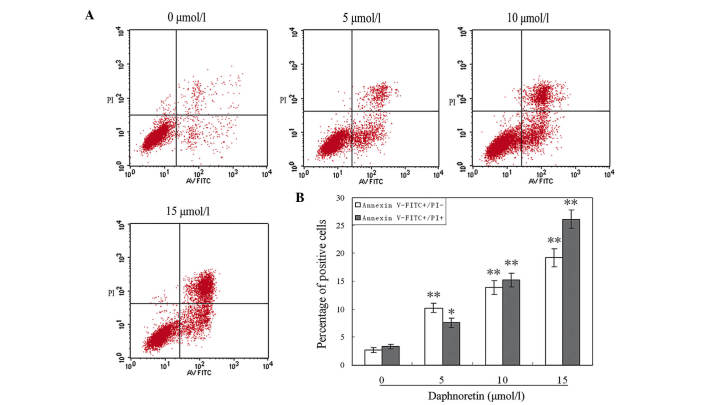
(A) Daphnoretin-induced apoptosis in A549 cells detected using flow cytometry. A549 cells were treated with daphnoretin (0, 5, 10 and 15 μmol/l) for 24 h. The cells were then harvested and stained with Annexin V and PI and flow cytometric analysis was performed to analyze apoptosis. (B) Summary of the apoptosis data. ^*^P<0.05 and ^**^P<0.01, vs. the respective control group. AV, avidin; FITC, fluorescein isothiocyanate; PI, ßpropidium iodide.

The ratio of apoptotic cells induced by daphnoretin was measured using flow cytometry. A549 cells were treated with various concentrations of daphnoretin (0, 5, 10 and 15 μmol/l) for 24 h and analyzed using flow cytometry with Annexin V and PI staining. As shown in Fig. 4, the ratio of early and late apoptotic cells was observed to be significantly increased in the daphnoretin-treated cells compared with the cells in the control group. The results show that when treated with daphnoretin for 24 h, the ratio of apoptotic cells significantly increased in a concentration-dependent manner.

### Effect of daphnoretin on Bcl-2 family gene expression

**Figure 5:**
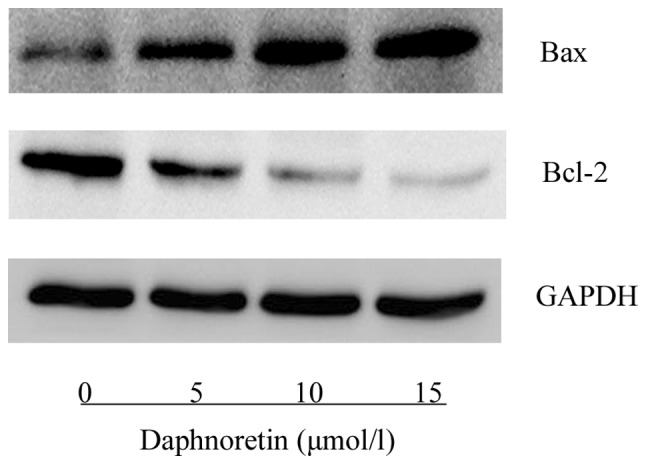
Effect of daphnoretin on Bcl-2 family protein expression detected using western blot analysis. A549 cells were treated with daphnoretin (0, 5, 10 and 15 μmol/l) for 24 h. Proteins were then extracted and Bax, Bcl-2 and GAPDH expression were analyzed using western blot analysis. Bcl, B-cell lymphoma; Bax, Bcl-2-associated X protein; GAPDH, glyceraldehyde-3-phosphate dehydrogenase.

The expression of apoptosis-related proteins was assessed using western blot analysis. As shown in Fig. 5, daphnoretin treatment induced an increase in Bax protein expression and a reduction in Bcl-2 protein expression compared with the control cells. The ratio of Bax to Bcl-2 was observed to increase in a concentration-dependent manner.

## Discussion

Increasing research attention has been focused on phytochemicals in the search for novel anticancer agents with enhanced efficacy to cancer cells and reduced toxicity to normal cells. Daphnoretin, isolated from *Wikstroemia indica* C.A. Mey. (16) as well as *Daphne mezereum* L. and *Daphne cannabina* Wall. (17), have been shown to induce cell cycle arrest and apoptosis in leukemia, osteosarcoma and HeLa cells (13–15). These findings suggest that daphnoretin may have potential as a novel anti-cancer agent.

In the present study, the results demonstrated that daphnoretin inhibited the growth of A549 lung cancer cells in a concentration- and time-dependent manner. A549 cells treated with daphnoretin exhibited typical apoptotic characteristics, including cytoplasmic shrinkage, plasma membrane blebbing, nuclear chromatin condensation, chromosomal DNA cleavage and fragmentation of the cells into membrane-enclosed vesicles or apoptotic bodies. Flow cytometric analysis revealed that daphnoretin induced A549 cell apoptosis in a concentration-dependent manner. Moreover, Bax/Bcl-2 were found to be involved in the molecular mechanism of daphnoretin-induced apoptosis in A549 cells.

Apoptosis is a type of programmed cell death which occurs through the activation of intrinsic cell suicide pathways (18). Apoptosis is a hallmark of anticancer drug-induced cell death (19). Proteins of the Bcl-2 family are key regulators of the apoptotic pathway (20,21). The Bcl-2 gene family, which is significantly involved in the regulation of cell apoptosis, includes anti-apoptotic genes, including Bcl-2 and Bcl-extra large and pro-apoptotic genes including, Bax, Bcl2-antagonist/killer, Bcl-2-interacting killer, BH3 interacting-domain death agonist and Bcl2-associated agonist of cell death (22,23). Certain traditional Chinese anticancer drugs have been found to induce cell apoptosis through targeting the proteins of the Bcl-2 family and the ratio of Bax/Bcl-2 (24,25). In the present study, western blot analysis revealed that the daphnoretin-induced apoptosis in the A549 cells was mediated through the downregulation of Bcl-2 protein expression and the upregulation of Bax protein expression.

In conclusion, the present study has demonstrated that daphnoretin is capable of inhibiting proliferation and promoting apoptosis in A549 lung cancer cells. Furthermore, this apoptotic response is associated with the regulation of the expression of the Bcl-2 gene family. These findings indicate that daphnoretin may have potential as a therapeutic agent for the management of lung cancer.
